# Genetic and biomarker approaches to uterine fibroids: toward precision medicine

**DOI:** 10.3389/fgwh.2025.1581823

**Published:** 2025-04-22

**Authors:** Pooja Mukherjee

**Affiliations:** ^1^ Innovative Genomics Institute, University of California, Berkeley, Berkeley, CA, United States; ^2^ Department of Molecular and Cell Biology, University of California, Berkeley, Berkeley, CA, United States

**Keywords:** uterine fibroid, biomarkers, precision medicine, leiomyoma, genetic predisposition, early diagnosis, women's health, quality of life

## Abstract

Uterine fibroids (UFs) are the most common benign tumors of the female reproductive system, affecting 70%–80% of women by age 50. Early detection is challenging due to the absence of initial symptoms, and diagnosis primarily relies on ultrasound and magnetic resonance imaging (MRI). However, biomarker-driven approaches could enable earlier and more precise detection. This review explores emerging biomarkers and genetic factors in fibroid pathogenesis. Potential biomarkers, including *PLP1, FOS, versican, LDH,* and *IGF-1*, show promise for diagnosis and recurrence prediction. Genetic studies have identified key mutations in *MED12, FH, HMGA2,* and *COL4A5-COL4A6*, alongside genome-wide association studies (GWAS) that highlight fibroid risk loci. Interestingly, biomarkers may also be mutation-type specific, suggesting potential for more precise molecular classification. Gene therapy offers an innovative treatment approach but the genetic landscape of fibroids remains underexplored, limiting advancements in research and funding. Integrating biomarker-based diagnostics and genetic profiling could transform fibroid detection and management, reducing reliance on invasive procedures. This review highlights the urgent need for improved diagnostic tools, prognostic markers, and targeted therapies for uterine fibroids.

## Uterine fibroids: a silent burden in women's health

Uterine fibroids (UFs), also known as leiomyomas or myomas, are benign tumors arising from the uncontrolled overgrowth of smooth muscle and connective tissue in the uterus. A hallmark of leiomyomas is the excessive deposition of a disorganized extracellular matrix (ECM), which normally provides structural support to tissues (1).

They are the most common benign tumors of the female reproductive system, affecting up to 70%–80% of women by the age of 50, with the highest prevalence occurring between 30 and 50 years of age. While some women remain asymptomatic, others experience a range of symptoms, including heavy menstrual bleeding, pelvic pain, frequent urination, and reproductive complications such as infertility or pregnancy loss. The symptoms often lead to limitations in daily activities, emotional distress and a reduced quality of life.

Despite their high prevalence, early diagnosis of fibroids is difficult due to the absence of detectable symptoms in the initial stages. Diagnosis is typically only possible after fibroids have grown large enough to be identified using imaging techniques such as ultrasound or MRI. Thyroid-stimulating hormone (TSH) (2) and prolactin levels (3) have been routinely used by gynecologists as part of the initial laboratory evaluation to exclude endocrine disorders that may mimic fibroid-related symptoms. While fibroids can occasionally coexist with thyroid nodules or, in rare cases, be associated with ectopic prolactin secretion, these hormones are not direct biomarkers of fibroids.

Fibroids are classified into four types based on their location within the uterus, each influencing symptoms differently (4):

• Intramural Fibroids: Most common type; they develop within the muscular wall of the uterus.
• Submucosal Fibroids: They are located just beneath the uterine lining and protruding into the uterine cavity; even small submucosal fibroids can lead to significant symptoms including heavy and prolonged menstrual bleeding.
• Subserosal Fibroids: They develop on the outer uterine surface and may exert pressure on surrounding pelvic organs.
• Pedunculated Fibroids: They are attached to the uterine wall by a stalk-like structure.

Treatment options for uterine fibroids vary based on factors such as size, location, severity of symptoms, and reproductive goals (4). Medications are used to manage symptoms by regulating hormones, reducing heavy menstrual bleeding, and shrinking fibroids. However, they do not eliminate fibroids completely. Minimally invasive procedures like uterine artery embolization (UAE) and MRI-guided focused ultrasound manage symptoms, while surgical options include myomectomy (fibroid removal while preserving the uterus) for fertility preservation and hysterectomy (complete removal of the uterus) as the only definitive cure. Although treatments through these procedures can be effective, recurrence rates remain a challenge. Studies suggest that 15%–33% of fibroids recur after myomectomy, with 10%–21% of women undergoing hysterectomy within five to ten years of myomectomy due to recurrence (5).

Approximately 40%–50% of fibroids exhibit chromosomal abnormalities, indicating a significant genetic component. Family history also plays a role; women with a first-degree relative affected by fibroids have a higher risk of developing them. Non-genetic factors influencing fibroid development include hormonal imbalances, obesity, and lifestyle choices. The age of onset and severity of symptoms can vary based on these factors. Genetically predisposed individuals may develop fibroids at a younger age and experience more pronounced symptoms, while those with non-genetic risk factors might encounter a later onset with varying symptom severity. Understanding the interplay between genetic and non-genetic factors is crucial for personalized management and treatment of uterine fibroids (1, 6–11). This review summarizes advancements in blood-based biomarkers for the diagnosis of uterine fibroids and explores the genetic factors contributing to their development. To ensure a comprehensive and up-to-date synthesis of the literature, a structured search was conducted using PubMed and Google Scholar, mainly focusing on studies published in the last 10–15 years. Search terms included combinations of “uterine fibroids”, “leiomyoma”, “genetic predisposition”, “biomarkers”, “early diagnosis”, and “non-surgical treatment”. Reference lists of key articles were manually screened to capture additional relevant studies. Studies were selected based on their relevance to genetic and non-genetic risk factors, biomarker research, and emerging therapeutic approaches.

## Advancements in biomarkers for the diagnosis of uterine fibroids

Efforts to identify reliable biomarkers for the diagnosis of uterine fibroids (UFs) are an ongoing process, aiming to improve early detection and develop molecular-based diagnostic tools that complement current imaging techniques. While ultrasound and MRI remain the clinical gold standards, molecular and genetic biomarkers will offer earlier diagnosis, enhanced prognostic accuracy, and personalized therapeutic strategies. Here, we describe potential biomarkers that could be used for the diagnosis of uterine fibroids.

*PLP1*, an X-linked gene encoding a major myelin protein in the central nervous system (CNS) is upregulated (12) and *FOS,* a non-X-linked gene encoding a transcription factor in the AP-1 complex is downregulated (13) at both mRNA and protein levels in uterine fibroid samples from Asian females undergoing myomectomy or hysterectomy. While the role of *PLP1* in uterine fibroids remains unclear, the downregulation of *FOS* has been linked to extracellular matrix (ECM) remodeling and fibrotic changes, suggesting its potential involvement in UF pathogenesis (12). Serum versican levels were also significantly lower in women with uterine fibroids compared to healthy controls in an Eastern Indian cohort (14). Since excessive ECM deposition is a hallmark of uterine fibroids, testing versican, a key ECM proteoglycan, could provide valuable diagnostic insights. Note, however, that the above studies were conducted in patients of different ethnicities, which may contribute to biomarker variability.

The successful identification of biomarkers would also facilitate the study of uterine fibroid recurrence after initial treatment. *LDH* and *IGF-1* are two such biomarkers whose protein levels decreased two days after uterus-preserving surgeries but increased again six months postoperatively. However, no clear correlation between fibroid recurrence and the upregulation of these markers was established, as testing did not extend beyond six months (15). Nonetheless, this approach holds promise for future studies on fibroid recurrence.

UFs are frequently associated with mutations in the mediator complex subunit 12 (MED12) gene, which plays a critical role in transcriptional regulation and cell proliferation. Given the involvement of *MED12* mutations in fibroid pathogenesis, biomarker profiles may vary based on mutation status. Biomarkers like *HPGDS* (hematopoietic prostaglandin D synthase) and *CBR3* (carbonyl reductase 3) may be specifically associated with *MED12*-mutated fibroids, suggesting that molecular classification could refine diagnostic and therapeutic strategies (16). *MED12*-mutant fibroids tend to be smaller but more numerous, with a rich extracellular matrix and poor vasculature, while wild-type fibroids exhibit higher vascularization and smooth muscle proliferation (17). These distinct phenotypic differences highlight the potential for personalized therapeutic strategies based on *MED12* mutation status.

Other technologies such as lipidomics provide additional diagnostic potential, with altered lipid profiles observed in UF patients (18). Urinary oxidative stress biomarkers have also been found to be upregulated in patients with fibroids, further supporting the role of metabolic changes in UF pathogenesis. Oxidative stress markers, such as lipid peroxidation (LOOH) products, advanced oxidation protein products (AOPPs), and carbonyl groups, are elevated in the sera of women with uterine fibroids, while antioxidant thiol levels are reduced, suggesting a role in fibroid development (17). Serum AOPP and carbonyl levels correlate with total fibroid weight and infertility duration, making them potential biomarkers for fibroid diagnosis and monitoring. Various other biomarkers have been investigated; however, no solid conclusions have been drawn (19).

These findings suggest that metabolic and molecular changes associated with fibroid development could be leveraged for novel diagnostic applications, particularly through blood plasma analysis. A systematic study involving diverse racial and age groups across different fibroid stages (no myoma, recurrent, and non-recurrent myoma), using both tissue and serum samples, is essential to identify a reliable and universally applicable biomarker for uterine fibroids.

## Uterine fibroid genetics

Uterine fibroid genetics can be classified into two categories: somatic mutations, which are genetic changes acquired during an individual's lifetime, and germline mutations, which are inherited variations passed down from parents that may significantly influence fibroid risk. Since ethnicity plays a major role in the occurrence and symptoms of uterine fibroids, understanding germline mutations is crucial for further exploration and the development of targeted therapies. Studies indicate that women who develop uterine fibroids before the age of 30 are more likely to have a genetic predisposition, as genetically influenced traits tend to manifest earlier in life (20, 21). In this regard, patients with familial fibroids tend to present with more severe symptoms, multiple fibroids, and high VEGF-A expression (22).

Although many such mutations have been identified, no systematic study has determined the extent of their heritability. Two most frequently mutated genes are *MED12* and Fumarate Hydratase (*FH*). With a frequency reported in the range of 50 to 85%, *MED12* is the most frequently mutated gene in uterine leiomyomas. *FH* is known to alter a tumor suppressor mechanism for uterine leiomyomata (23). UF patients with *FH* mutations have an increased risk of renal cancer (24). Genetic predisposition to increased fat-free mass was found to be causally linked to a higher risk of uterine fibroids. Genes within the myocardin and cyclin-dependent kinase inhibitor 1A pathways involved in smooth muscle cell differentiation and proliferation appear to play a role in fibroid development (25). Other mutated genes include *HMGA2* (encoding a high mobility group protein involved in transcriptional regulation) *and COL4A5-COL4A6* (X-linked gene, encoding collagen proteins) genes (26)*.*

Studies show that treatment response can vary based on the genetic subclass of uterine fibroids. For example, *MED12* mutant fibroids are 4.4 times more likely to shrink than *HMGA2* driven fibroids, highlighting the importance of genetic profiling in personalized fibroid treatment (27, 28).

Genome-wide association studies have aimed to identify genetic risk loci associated with uterine fibroids. One such study found shared genetic origins between uterine leiomyomata and endometriosis (29). However, for these findings to be truly valuable, risk loci must be identified across diverse ancestral backgrounds (30), the aberrant regulation of risk loci in uterine fibroids must be validated to explain the disease phenotype, and the identified genetic targets must be tested *in vivo* using cell line models or mouse models. In this regard, Buyukcelebi et al. advanced the field by integrating genetic risk loci with bulk and single-cell gene expression data, as well as serum protein levels, to identify potential molecular traits downstream of genetic risk loci. Their study found that in leiomyoma samples, smooth muscle cells, the cell type of origin for uterine fibroid tumors, contained the highest number of highly expressed GWAS target genes, with 40 percent of differentially expressed GWAS targets being selectively enriched in these cells.

A fundamental question remains regarding whether these genetic mutations are inherited or acquired during a person's lifetime. Chromosomal abnormalities are frequently observed in uterine fibroids (1, 24), further complicating the classification of genetic changes in fibroid development. Several other genes have also been linked to uterine leiomyoma development, and a multi-ethnic study identified fourteen genetic loci that may influence fibroid predisposition (7). These genes fall into three major categories: those involved in telomere length regulation, those involved in DNA damage response mechanisms, and those playing a role in genitourinary system development. Mutations or dysregulation in these genes may contribute to fibroid onset and progression.

A family history of fibroids does not necessarily guarantee that an individual will develop them, as hormonal factors also play a significant role. Both estrogen and progesterone directly stimulate fibroid growth, which becomes particularly evident during pregnancy when elevated hormone levels can trigger rapid fibroid expansion (31). Future research should focus on determining whether a common mechanistic link exists between various genetic alterations and hormonal pathways driving fibroid formation. Such findings could pave the way for targeted, personalized therapies that integrate genetic screening, biomarker-based diagnostics, and hormonal regulation strategies for effective fibroid management.

## Gene therapy

Gene therapy presents an experimental but promising alternative with the potential for a lasting cure (32). To date, two main strategies have been explored in preclinical studies for uterine fibroids. The first involves suppressing the estrogen receptor in uterine fibroid cells, thereby reducing estrogen binding and receptor activity, which leads to decreased fibroid cell proliferation (11, 33, 34). The second approach utilizes targeted delivery of the herpes simplex virus thymidine kinase (HSV-TK) gene, followed by treatment with ganciclovir (GCV), which selectively induces cell death in fibroid cells (35).

One of the major challenges in developing gene therapy for uterine fibroids is identifying the appropriate target gene. This limitation may be addressed by conducting *in vivo* studies to identify and validate causal genes across patients of different racial backgrounds. While emerging research has begun identifying potential targets as mentioned above, these findings remain preliminary and require further validation.

On the other hand, a notable advantage of fibroid gene therapy is the potential for selective gene delivery to fibroid tissue. Once an appropriate target gene is identified, gene therapy could emerge as a viable therapeutic option. However, its clinical application remains distant, primarily due to the complex and less-explored genetic landscape of the disease, as well as the non-life-threatening nature of fibroids, which has limited research funding and focus.

Nevertheless, it is important to acknowledge the substantial impact fibroids can have on younger women's lifestyle and mental health. Living with fibroids often involves undergoing multiple medical procedures, extended recovery periods, and the emotional distress of recurrence, all of which can significantly affect quality of life (36, 37).

## Discussion

Currently, uterine fibroids are diagnosed either during routine check-ups or when a patient presents with symptoms. By the time they are detected, fibroids have often already begun growing, initiating a treatment journey that primarily focuses on symptom management (38). In most cases, surgical treatment options eventually lead to either myomectomy (removal of fibroids while preserving the uterus) or hysterectomy (removal of the uterus) for long-term relief. For many women, this means facing difficult decisions, such as planning a family earlier than desired to avoid fibroid recurrence or, in severe cases, undergoing a hysterectomy at a young age. Early genetic screening, particularly for individuals with a family history of fibroids, may help assess the risk of fibroid development and enable earlier interventions. Additionally, the identification of serum and menstrual blood biomarkers has shown promise in research settings, but further validation is required before they can be adopted for routine early diagnosis and that could significantly advance the field of early fibroid diagnostics. While genetic predisposition plays a key role in the development of uterine fibroids, environmental and lifestyle factors such as vitamin D deficiency, dietary patterns and obesity may interact with genetic susceptibility to influence fibroid growth and severity. Understanding these factors allows for a more comprehensive view of fibroid pathogenesis and potential prevention strategies. Studies suggest that simple, non-invasive treatments, such as hormonal pills or addressing Vitamin D deficiency, may help slow fibroid progression if initiated early (39–42) but more clinical evidence is needed to support these preventative strategies. If such preventive measures are implemented from a young age, many women may be able to avoid surgery altogether. For those who do not respond to conventional treatments, gene therapy could offer a promising long-term solution. Analyzing fibroids removed during surgery may help identify mutational burden and could contribute to the development of preventive vaccines to reduce recurrence in future. However, this idea remains highly speculative and is not currently supported by clinical trials.

The ability to make reproductive choices without the looming pressure of fibroid-related complications would significantly alleviate the mental and emotional burden that many young women currently face. A crucial first step in this direction would be a comprehensive study of different ethnic populations to identify differentially expressed mRNAs and proteins in uterine fibroids. Such research could help uncover common fibroid pathogenesis pathways, potentially leading to targeted and personalized treatments. Breakthroughs in this area could mirror those seen in other complex disease fields (43).

**Figure 1:**
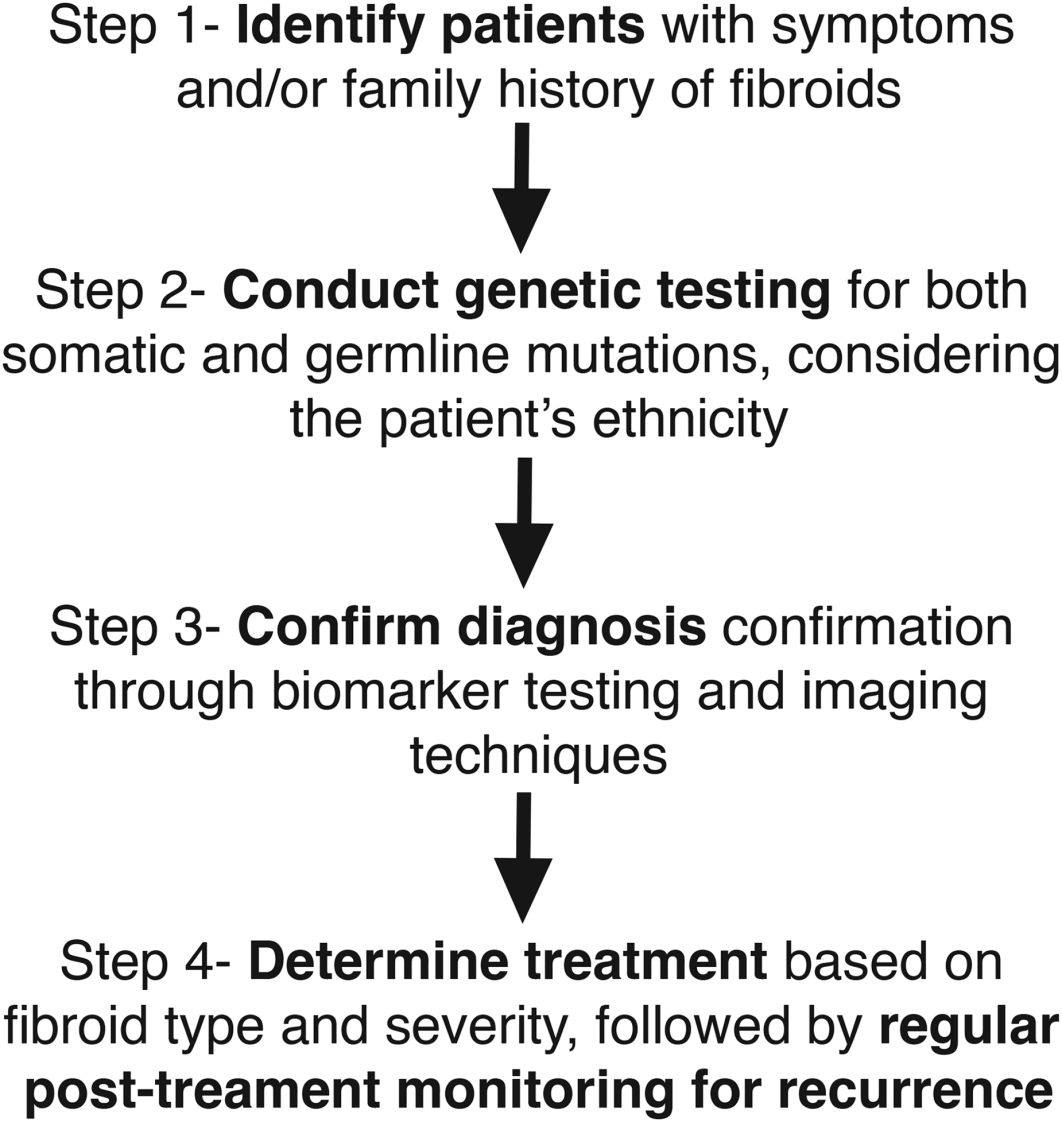
Integration of biomarkers and genetics in fibroid diagnosis: A proposed model. This figure illustrates a stepwise model for integrating biomarkers and genetic testing into the diagnosis and management of uterine fibroids. Step 1 involves identifying patients based on symptoms and/or family history of fibroids. Step 2 consists of genetic testing for both somatic and germline mutations, taking into account ethnic variations. Step 3 focuses on confirming the diagnosis through biomarker testing and imaging techniques. Step 4 determines treatment based on fibroid type and severity, with regular post-treatment monitoring for potential recurrence. This model highlights a personalized approach to fibroid diagnosis and management by incorporating genetic and biomarker-based strategies.

Recent advancements in gene editing techniques have enabled the development of cellular and animal models for studying uterine fibroids (30, 44). Additionally, multiple ongoing mechanistic studies are shedding light on fibroid development and progression (27, 45–48). The majority of ongoing clinical trials focus on interventional treatments, yet a significant number have been terminated over the years. One of the main reasons for trial failure is poor patient accrual, indicating difficulty in recruiting enough suitable participants. This highlights the need for a more refined classification of uterine fibroid patients based on fibroid pathophysiology to ensure that treatments are tailored accordingly (Figure 1) and that clinical trials target specific patient subgroups for better outcomes.
